# Yield of brain MRI in children with autism spectrum disorder

**DOI:** 10.1007/s00431-023-05011-2

**Published:** 2023-05-25

**Authors:** D Byrne, A Fisher, L Baker, EL Twomey, K M Gorman

**Affiliations:** 1 Department of Neurodisability, Children’s Health Ireland at Temple Street, Dublin 1, Ireland; 2 Department of Radiology, Children’s Health Ireland at Temple Street, Dublin 1, Ireland; 3 Department of Paediatric Neurology and Clinical Neurophysiology, Children’s Health Ireland at Temple Street, Dublin 1, Ireland; 4 School of Medicine and Medical Sciences, University College Dublin, Dublin 4, Ireland

**Keywords:** Autism, ASD, Neurodevelopment, Neuroimaging, MRI

## Abstract

Autism spectrum disorder (ASD) is a common neurodevelopmental condition. The American Academy of Paediatrics and American Academy of Neurology do not recommend routine brain magnetic resonance imaging (MRI) in the assessment of ASD. The need for a brain MRI should be decided on atypical features in the clinical history and examination. However, many physicians continue to use brain MRI routinely in the assessment process. We performed a retrospective review of indications for requesting brain MRI in our institution over a 5-year period. The aim was to identify the yield of MRI in children with ASD and calculate the prevalence of significant neuroimaging abnormalities in children with ASD and identify clinical indications for neuroimaging. One hundred eighty-one participants were analysed. An abnormal brain MRI was identified in 7.2% (13/181). Abnormal brain MRI was more likely with an abnormal neurological examination (OR 33.1, *p* = 0.001) or genetic/metabolic abnormality (OR 20, *p* = 0.02). In contrast, abnormal MRI was not shown to be more likely in children with a variety of other indications such as behavioural issues and developmental delay.

*      Conclusion*: Thus, our findings support that MRI should not be a routine investigation in ASD, without additional findings. The decision to arrange brain MRI should be made on a case-by-case basis following careful evaluation of potential risks and benefits. The impact of any findings on the management course of the child should be considered prior to arranging imaging.

**Table:** 

**What is Known:** *• Incidental brain MRI findings are common in children with and without ASD.* *• Many children with ASD undergo brain MRI in the absence of neurological comorbidities.*	
**What is New:** *• Brain MRI abnormalities in ASD are more likely with an abnormal neurological examination and genetic or metabolic conditions.* *• Prevalence of significant brain MRI abnormalities in ASD alone is low.*

**What is Known:**

*• Incidental brain MRI findings are common in children with and without ASD.*

*• Many children with ASD undergo brain MRI in the absence of neurological comorbidities.*

**What is New:**

*• Brain MRI abnormalities in ASD are more likely with an abnormal neurological examination and genetic or metabolic conditions.*

*• Prevalence of significant brain MRI abnormalities in ASD alone is low.*

## Introduction

Autism spectrum disorder (ASD) is a neurodevelopmental disorder characterised by persistent deficits in social communication and social interaction and restricted, repetitive patterns of behaviour, interests, and activities. To meet the diagnostic criteria for ASD according to DSM-5, a child must have persistent deficits in each of the three areas of social communication and interaction plus at least two of four types of restricted, repetitive behaviours [1]. There is a spectrum of cognitive abilities within this definition, from severe intellectual disability (ID) to average or above average intelligence quotient (IQ). Worldwide the prevalence of ASD is variable ranging from 0.7 to 2.9% depending on the cohort studied (age, sex, location, and study period) [2–4]. The estimated prevalence of ASD in Ireland is approximately 1.5% in a school population aged 6–11 years [5]. The prevalence has increased especially in the past 20 years due to a combination of factors, including greater public awareness, more accurate diagnostic criteria, and changes in the diagnostic criteria [4].

Guidance from the American Academy of Pediatrics (AAP) and the American Academy of Neurology (AAN) suggests routine brain magnetic resonance imaging (MRI) is not part of the initial evaluation or diagnosis of ASD [6, 7]. According to the AAP, approximately a quarter of children with ASD may show signs of developmental regression in language or social skills, which is not likely attributable to an underlying neurodegenerative cause. The need for a brain MRI should be decided based on the clinical history and examination. Neuroimaging may be indicted in the evaluation of atypical regression, microcephaly, macrocephaly, seizures, intracranial manifestations of genetic disorders, abnormal neurological examination, or other indications [6].

Previous studies have estimated the yield of MRI in ASD to be variable, and often findings not related to the presentation and not helpful in diagnostic work-up [7–9]. However, these studies were population-specific and only evaluated an association between limited reasons for referral (ASD alone vs ASD and epilepsy, abnormal neurology, headache, microcephaly, and macrocephaly) [8, 9]. Frequently in clinical practice, brain MRI is requested due to a diagnosis of ASD without additional features. Therefore, the aim of this retrospective review was to identify the yield of MRI in children with ASD and calculate the prevalence of significant neuroimaging abnormalities in children with ASD with and without a variety of other indications, including those not previously studied. This will provide additional information for clinicians who evaluate children with ASD.

## Methods

### Study design

We undertook a retrospective review of indications for brain MRI in children < 16 years in Children’s Health Ireland at Temple Street, Dublin, from January 1, 2015, to December 31, 2019. The radiology requesting system (Philips XIRIS) was searched using keywords (ASD, autism, autistic and spectrum). Children’s Health Ireland at Temple Street is a tertiary referral centre, with facilities to perform MRI under general anaesthetic.

#### Inclusion criteria

Children under 16 years of age with confirmed or suspected ASD, with and without other reasons for referral, undergoing brain MRI for investigation of ASD.

#### Exclusion criteria

Children with a previously known parenchymal lesion or diagnosed neurodegenerative disorder neuroimaging performed for an acute neurological condition such as meningitis and/or older than 16 years.

### Brain MRI findings

All brain MRI images were acquired on a single 1.5-T General Electric HDxt SIGNA scanner. Neuroimaging findings were separated into two groups: *normal* and *abnormal.* All abnormal neuroimaging was reviewed by paediatric radiologist (ET). *Abnormal findings* were defined as white or grey matter signal abnormalities and structural malformations. Incidental findings were defined as radiological observations unrelated to the purpose of the examination and unlikely to have a clinical impact or significance were included in the *normal* group [10]. Examples of incidental findings include arachnoid cyst, developmental venous abnormality, or mucosal thickening.

### Statistical analysis

Data was analysed using the SPSS version 26.0 (IBM SPSS Statistics, IBM Corporation). Frequencies and percentages were calculated to compare groups. Statistical significance was determined at* P* value less than 0.05. Binary logistic regression was employed to analyse the relationship between developmental delay, epilepsy, behavioural problems, abnormal neurology, metabolic or genetic abnormality, ASD alone, macrocephaly, atypical regression, high risk history, microcephaly, headache/vomiting, and other abnormal MRI. Chi-square test was used to calculate *p* values for baseline characteristics.

### Ethical approval

Ethical approval was granted by the local research and ethic committee (ref: CA1912-02).

## Results

**Fig. 1:**
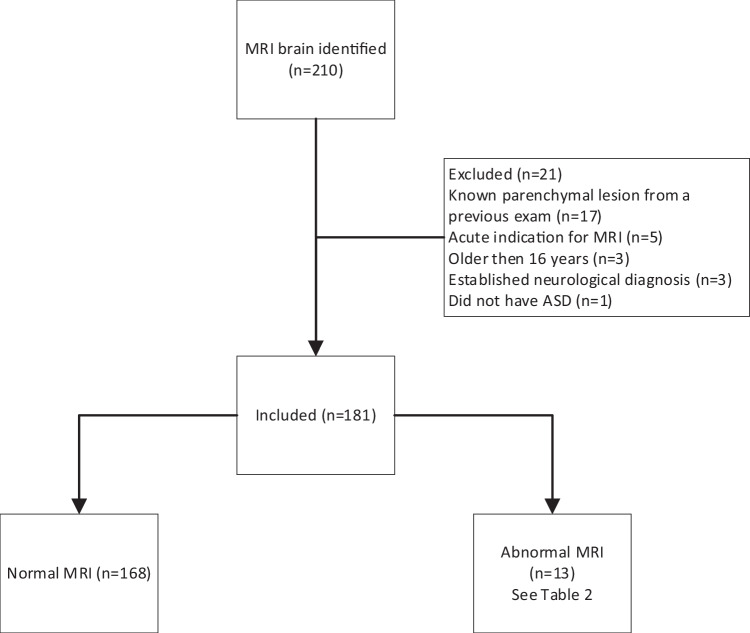
Flowchart of patient enrolment

We identified 210 brain MRI examinations using the search terms. There was a total of 7177 brain MRI exams performed during the same period. Twenty-nine patients were excluded and 181 were included in the analysis (Fig. 1).

Participant baseline characteristics are described in Table 1. The median age at the time of neuroimaging was 7.9 years (IQR 5.3–11.7 years). General paediatrics was the most common referring specialty (53%, 95/181), followed by paediatric neurology (32%, 57/181).

**Table 1:** Baseline characteristics

	**Total** ***n* (%)**	**Normal** ***n* (%)**	**Abnormal** ***n* (%)**	***p* value**
**Total**	181	168 (93)	13 (7)	-
**Age**				
** 1–6 years**	62 (34)	55 (89)	7 (11)	0.12
** 6–10 years**	60 (33)	58 (97)	2 (3)	0.16
** 10–16 years**	59 (33)	55 (93)	4 (7)	0.88
**Gender**				
** Male**	151 (83)	139 (92)	12 (8)	0.39
** Female**	30 (17)	29 (97)	1 (3)	0.37
**Referral source**				
** General Paediatrics**	95 (52)	88 (93)	8 (7)	0.5
** Neurology**	57 (31)	56 (98)	1 (2)	0.055
** Metabolic medicine**	18 (9.9)	15 (83)	3 (17)	0.1
** Neuro-disability**	8 (4.4)	8 (100)	0 (0)	0.42
** Other**	3 (1.7)	2 (67)	1 (33)	
**Sedation required**				
** General anaesthesia**	155 (86)	143 (92)	12 (8)	0.48
** Conscious sedation**	13 (7.2)	13 (100)	0 (0)	0.3
** Walk-in**	12 (6.6)	11 (92)	1 (8)	0.94

### Indications for imaging

All indications were recorded (*n* = 28), with majority of the children (66%, 119/181) having greater than one indication listed on the imaging request. Developmental delay (38%, 69/181) and epilepsy (29%, 52/181) associated with ASD were the most common indications followed by behavioural issues (19%, 35/181) and abnormal neurological examination (9.9%, 18/181) (Table 2).

**Table 2:** Binary logistic regression analysis indications for MRI

	**Total** ***n* (%)**	**Normal** ***n* (%)**	**Abnormal** ***n* (%)**	**Odds ratio** **(95% CI)**	***p* value**
**Indication**					
**Developmental delay**	69 (22)	62 (90)	7 (10)	3.85 (0.9–17)	0.07
**Epilepsy**	52 (29)	49 (94)	3 (6)	4.18 (0.6–31)	0.16
**Behavioural issues**	35 (19)	33 (94)	3 (6)	0.36 (0.05–2.6)	0.31
**Abnormal neurological examination**	18 (9.9)	13 (72)	5 (28)	33.4 (4.4–251)	0.001
**Metabolic or genetic abnormality** ^**a**^	16 (8.8)	12 (75)	4 (25)	20 (2.9–138)	0.02
**ASD alone**	11 (6.0)	11 (100)	0	0	0.99
**Macrocephaly**	11 (6.0)	11 (100)	0	0	0.99
**Atypical regression**	11 (6.0)	11 (100)	0	0	1
**High risk history **^**b**^	10 (5.5)	9 (90)	1 (10)	2.3 (0.2–26)	0.5
**Microcephaly**	8 (4.4)	7 (87)	1 (13)	7.1 (0.5–97)	0.14
**Headache and/or vomiting**	6 (3.3)	6 (100)	0	0	1
**Other**	26 (14)	26 (100)	0	0	1

^a^Suspected or confirmed

^b^Prematurity, perinatal asphyxia, meningitis, and non-incidental injury

### Brain MRI results

The prevalence of abnormal brain MRI was 7.2% (13/181) (Table 1). Significant findings identified included white matter signal abnormalities, volume loss, cortical heterotopia, and optic atrophy (Table 3). Incidental findings were noted in 22% (39/181) including mucosal sinus opacification, enlarged Virchow Robin perivascular spaces, choroid fissure cysts, arachnoid cysts, and Rathke cleft cysts.

**Table 3:** MRI abnormalities

*MRI finding*	*Indication for scan (in addition to ASD)*
Increased T_2_ signal in the globus pallidi and dentate nuclei bilaterally	Developmental delay and laboratory findings suggesting metabolic disease (succinic semialdehyde dehydrogenase deficiency)
Several small focal areas of T_2_ hyperintensity in deep white matter	Developmental delay and epilepsy
Asymmetric high T_2_ and FLAIR signal intensity in the deep white matter posteriorly	Laboratory evidence of a genetic disorder
Subependymal and adjacent white matter cortical heterotopia	Developmental delay, obesity, and confirmed genetic neurodevelopmental disorder
Patchy white matter T_2_ hyperintensity on a background of hypomyelination	Developmental delay and confirmed metabolic disease
Porencephalic cyst in adjacent to the body of the right lateral ventricle and periventricular white matter volume loss with T_2_ hyperintensity	Prematurity, intraventricular haemorrhage, abnormal neurological examination (focal spastic weakness)
Defect in medical aspect of occipital lobe with associated volume loss, consistent with prior infarct	Epilepsy
Scattered foci of T_2_/FLAIR hyperintensity in both cerebral hemispheres and adjacent to the posterior horns of both lateral ventricles	Neurocutaneous stigmata
Minor increased T_2_ FLAIR signal within the posterior periventricular white matter	Abnormal neurological examination (ataxia)
Progressive left optic nerve atrophy	Epilepsy and abnormal neurological examination (left optic atrophy)
Mild optic nerve atrophy, cerebellar tonsillar ectopy. Diffuse thickening of skull bone. Effacement of CSF cisterns	Developmental delay, vision loss
Areas of T_2_/FLAIR signal abnormality adjacent to the posterior horns of the lateral ventricles bilaterally, with mild associated volume loss	Developmental delay, poor social contact, behavioural issues
Multifocal small regions of parenchymal volume loss and signal abnormality	Microcephaly, developmental delay, behavioural issues

In children with only ASD as the indication for neuroimaging, there was no pathology identified (11/181). Abnormal neurological examination (OR 33.1 (4.4–251), *p* = 0.001) and genetic/metabolic abnormality (OR 20 (2.9–138), *p* = 0.02) were associated with abnormal brain MRI. Abnormal genetic/metabolic abnormality includes abnormal array -CGH (*n* = 2) and metabolic de-arrangements (raised lactate, high urine 3-hydroxybutyrate, and classical galactosemia). Other indications did not have a statistically significant association with abnormal brain MRI (Table 2).

## Discussion

In our retrospective review of neuroimaging performed in the evaluation of ASD, abnormal brain MRI was identified in 7.2% (13/181). Abnormal brain MRI was more likely with an abnormal neurological examination or genetic/metabolic abnormality. In contrast, abnormal MRI was not shown to be more likely in children with a variety of other indications such as behavioural issues and developmental delay. Thus, our findings support that MRI should not be a routine investigation in ASD, without additional findings.

We identified a 7.2% (13/181) prevalence of abnormal MRI in our cohort which is consistent with previous studies. We analysed incidental findings (39/181) under the *normal* group, as the findings were not related to presentation. Cooper and colleagues found a 6.5% prevalence of pathology in patients whose MRI request indication was ASD alone [9]. The prevalence was highest in those with ASD and an abnormal neurological examination or pre-existing finding (26%) [9]. This mirrors our cohort in which abnormal neurological examination and abnormal genetic/metabolic laboratory findings were associated with abnormal MRI findings. Ming and colleagues found a prevalence of 15% of abnormal findings but included incidental findings, which was defined as normal in our study [8]. A recent paper by Rochat and colleagues of 117 children with ASD reported an abnormal brain MRI in 55% and minor abnormalities accounted for approximately one-third of the abnormal findings [11].

When extrapolating data on abnormal brain MRI, clinicians need to be cognisant if incidental findings are defined as abnormal or normal in analysis. There is a high rate of incidental findings on brains MRI in the general paediatric population. In our cohort, the prevalence of incidental findings was 22%. Studies vary but prevalence of up to one-third has been reported [10, 12, 13]. Incidental finding rates between children with ASD and neurotypically developing children appear to be similar, 5.5–68% [14–16]. However, the incidental finding rate in children with ASD may be higher [11, 17]. Thus, when ordering a brain MRI scan for a child with ASD, families need to be counselled about the higher frequency of incidental findings, which may cause extra anxiety for family and the need to follow-up neuroimaging.

Abnormal MRI findings included white matter signal abnormalities (*n* = 6) and/or volume loss (*n* = 4). As ASD does not have known MRI features, these findings are not diagnostic or suggestive of ASD but rather may indicate a prior injury or an underlying neurological condition. White matter abnormalities have been identified in other ASD cohort but also in developmental delay. Abnormalities, even if radiologically significant, may be clinically incidental [11, 18]. Other groups have hypothesised that white matter signal abnormalities may affect brain connectivity and supported by diffusion tensor imaging techniques [17, 19]. There may be a future diagnostic role for the use of advance neuroimaging techniques, as previous studies have reported changes in resting state functional connectivity, brain metabolism, neurotransmission, and neuroinflammation in adult patients compared to controls [20]. However, much is still to be understood about the genetics, brain connectivity, and brain development in ASD.

**Fig. 2:**
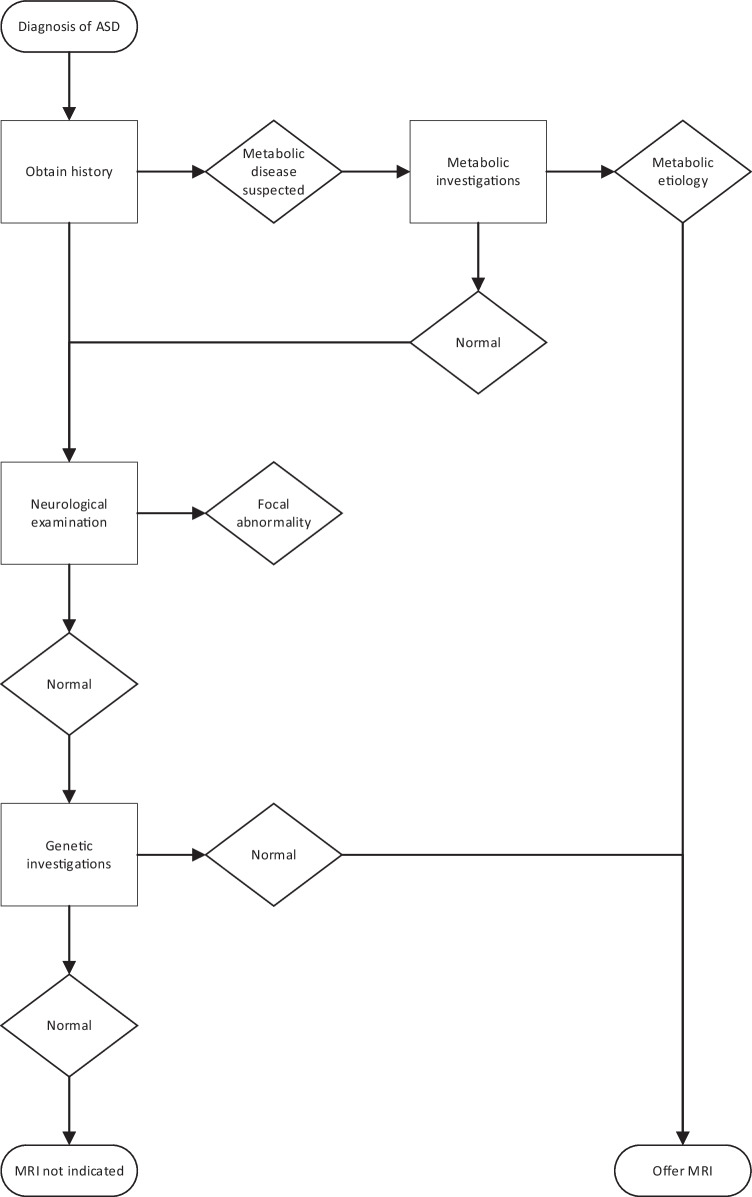
Suggested algorithm for brain MRI in ASD

Although guidelines exist on the role of brain MRI in children with ASD [3, 4, 21], commonly brain MRIs are performed, which are not indicated and do not contribute to the management or prognostication. As highlighted in our cohort, AAP guidelines were not followed when requesting scans [6]. Behavioural issues (19%, 35/181) and developmental delay (22%, 69/181) were the most common listed indications on MRI requests, despite these being common and typical features of ASD. In our cohort, children with ASD alone (11/181), all had normal neuroimaging. Thus, our findings support that brain MRI should not be a routine investigation in ASD, without additional findings. Our data showed an association between abnormal brain MRI and abnormal neurological examination and genetic/metabolic abnormality. Genetic investigations should be routinely offered to children with ASD as per AAP recommendations and in selective cases metabolic investigations [6]. If these reveal abnormalities, neuroimaging should be considered and is supported by our data. Figure 2 is a suggestive algorithm for selecting ASD patients for brain MRI. This has led to a change of policy in our radiology department, with the data from this study to reject requests which do not meet criteria.

Brain MRI may be required in other cases; however, the presence of ASD should not directly influence the decision to perform imaging. For example, in a child with ASD and focal or refractory epilepsy, brain MRI may modify the management of epilepsy, but it is not likely to affect the management of ASD. Of note, in our cohort, 52 had MRI for ASD and epilepsy and 3 were abnormal (6%). Abnormal findings included white matter abnormalities, optic atrophy and previous infarct.

Most children with ASD require a general anaesthetic to successfully carry out a brain MRI exposing them to anaesthesia related risks, and while this risk is minimal in children, it may be significant in toddlers [22]. In addition, the process may be stressful for children with ASD (combination of sensory issues, anxiety, poor communication skills) and for their families.

Our study has a number of limitations. As we studied children with ASD, we were not able to determine the prevalence of similar brain MRI findings in our general child population. The significance of brain MRI findings was determined based on the opinion of the reporting radiologist which may be subjective. The indications for neuroimaging were recorded from radiology requests, logged in free text. Indications not listed in the request were presumed absent. This may be sensitive to documentation bias. The degree of severity of some presentations such as developmental delay could not be determined.

## Conclusion

Our study did not show a yield for MRI in the routine investigations of ASD without high-risk clinical features which suggest an underlying diagnosis. The decision to arrange brain MRI should be made on a case-by-case basis following careful evaluation of potential risks and benefits. The impact of any findings on the management course of the child should be considered prior to arranging imaging.

## Data Availability

Data can be provided on request.

## Abbreviations

**AAN**: American Academy of Neurology
**AAP**: American Academy of Pediatrics
**ASD**: Autism spectrum disorder
**ID**: Intellectual disability
**IQ**: Intelligence quotient
**IQR**: Interquartile range
**MRI**: Magnetic resonance imaging
**OR**: Odds ratio
